# Behavior of Molybdenum–Vanadium Mixed Oxides in Selective Oxidation and Disproportionation of Toluene

**DOI:** 10.3390/ma12050748

**Published:** 2019-03-05

**Authors:** Gheorghita Mitran, Florentina Neaţu, Octavian D. Pavel, Mihaela M. Trandafir, Mihaela Florea

**Affiliations:** 1 University of Bucharest, Department of Organic Chemistry, Biochemistry & Catalysis, Faculty of Chemistry, 4-12, Blv. Regina Elisabeta, 030018 Bucharest, Romania; octavian.pavel@chimie.unibuc.ro; 2 National Institute of Material Physics, 405A Atomiştilor, PO Box MG 7, 077125 Măgurele, Romania; florentina.neatu@infim.ro (F.N.); mihaela.trandafir@infim.ro (M.M.T.)

**Keywords:** molybdena–vanadia, toluene disproportionation, toluene oxidation, p-xylene, benzaldehyde

## Abstract

This study deals with the behavior of molybdenum–vanadium (Mo/V) mixed oxides catalysts in both disproportionation and selective oxidation of toluene. Samples containing different Mo/V ratios were prepared by a modified method using tetradecyltrimethylammonium bromide and acetic acid. The catalysts were characterized using several techniques: nitrogen adsorption–desorption isotherms, X-Ray diffraction (XRD), ammonia temperature-programmed desorption (TPD-NH_3_), temperature-programmed reduction by hydrogen (H_2_-TPR), X-ray photoelectron spectroscopy (XPS), Raman spectroscopy, Fourier-transform infrared-spectroscopy (FTIR) and ultraviolet-visible spectroscopies (UV–VIS). The XRD results evidenced the presence of orthorhombic α-MoO_3_ and V_2_O_5_ phases, as well as monoclinic β-MoO_3_ and V_2_MoO_8_ phases, their abundance depending on the Mo to V ratio, while the TPD-NH_3_ emphasized that, the total amount of the acid sites diminished with the increase of the Mo loading. The TPR investigations indicated that the samples with higher Mo/V ratio possess a higher reducibility. The main findings of this study led to the conclusion that the presence of strong acid sites afforded a high conversion in toluene disproportionation (Mo/V = 1), while a higher reducibility is a prerequisite to accomplishing high conversion in toluene oxidation (Mo/V = 2). The catalyst with Mo/V = 1 acquires the best yield to xylenes from the toluene disproportionation reaction, while the catalyst with Mo/V = 0.33 presents the highest yield to benzaldehyde.

## 1. Introduction

Disproportionation of toluene is an important route to produce xylenes, as valuable intermediates for the production of more added value polymeric materials, such as polyesters or polyethylene terephthalates (PET) [1,2]. Therefore, this process has been intensively studied over the years using different zeolites as catalysts [3,4,5,6,7]. These studies concluded that the para-selectivity is influenced by the silicon to aluminum ratios. According to a recent report by Corma et al. [7] a high Si/Al ratio is not only used to reduce the acidity but to provide an environment able to accommodate the transition states of this reaction. Despite the fact that the active species for this reaction has been generally considered to be Brønsted acid site [8], a synergy effect of Brønsted acid sites and Lewis acid sites cannot be ruled out [9]. Moreover, the addition of La_2_O_3_ led to a decrease in the acidity of ZSM-5 [10] and also of the activity, but the reaction took place with an enhancement in the p-xylene yield. A similar behavior has been reported for the core/shell-structured borosilicate, **M**-t**W**enty-t**W**o (MWW)-type zeolites [1], where the presence of boron induced the creation of active sites required for such reactions. There are also reports, where the change of the environment, as in the case of the mordenite catalyst [11], induces the decrease of the activity merely caused by an enhanced coke production leading to pore blockage.

Replacing Al and Si in mixed oxides with other cations, provides another class of acid catalyst able to carry out the disproportionation. Accordingly, tungsten–niobium oxides [12] present a higher catalytic activity compared to tungsten–zirconium oxides, mordenite or silica–alumina [13,14]. Moreover, it was shown that addition of Mo in the catalyst for toluene conversion facilitated the disproportionation reaction in the detriment of hydrodealkylation and hydrocracking and has the ability to increase the resistance to deactivation of the catalysts [15,16].

On the other side, the selective oxidation of toluene is another reaction largely investigated by metal oxides. The production of benzaldehyde from toluene is still challenging, taking into account the fact that benzaldehyde is an important intermediate in the manufacture of perfumes and pharmaceuticals [17]. However, to date, it is still produced via a non-green procedure involving the toluene chlorination and hydrolysis of chlorinated derivates, with the disadvantage of a large volume of wastes [18]. Therefore, the selective oxidation starting directly from toluene, in the presence of heterogeneous catalysts, is preferable since in this case, water is the only side product.

Most of the studies devoted to toluene catalytic transformation focused on the use of vanadium as an active element, and among the various modified vanadia heterogeneous catalysts, V–Ag–O complex oxides presented both a good conversion of toluene and a high selectivity to benzaldehyde. These results were interpreted as a consequence of a reduced surface acidity and redox ability generated by the introduction of Ag into the VO_x_ structure [19]. In addition, V–Zr–O complex oxides [20] provided a good selectivity in the oxidation of toluene that has been associated to the structure of the incorporated ZrV_2_O_7_ or V–O–Zr phases. Ti-sepiolite supported vanadium oxide [21] is another example of the catalytic activity enhanced, showing the role of the density of the active sites.

In the past, molybdenum–vanadium mixed oxides have been reported as efficient catalysts for the oxidative dehydrogenation of alkanes [22,23], oxidation of acrolein to acrylic acid [24] or the total oxidation of aromatic hydrocarbons [25]. The catalytic activity of these mixed oxides was found to be strongly dependent on the oxide capability to generate surface oxygen species [26]. Moreover, it was established that the materials based on vanadium and molybdenum oxides exhibit interesting activity in the generation of the singlet form of molecular oxygen [27].

In line with the depicted state-of-the-art, in this study, we decided to investigate molybdenum–vanadium mixed oxides with the aim to understand the structure–activity relationships and to identify the acidic and redox properties by testing them in disproportionation and selective oxidation of toluene, respectively. For this purpose, mixed oxides with different Mo/V molar ratios were prepared by a modified sol-gel method, thermally treated at different temperatures and characterized by different techniques.

## 2. Materials and Methods

### 2.1. Catalysts Preparation

The reagents used in the present work were tetradecyltrimethylammonium bromide (from Aldrich, St. Louis, MO, USA, 99% purity), (NH_4_)_6_Mo_7_O_24_·4H_2_O (Fluka Analytical, Buchs, Switzerland, 98% purity), NH_4_VO_3_ (from Aldrich, St. Louis, MO, USA, 99% purity) and acetic acid (Chimactiv, Bucharest, Romania, 99.5% purity).

Catalysts with molar ratios of Mo/V = 0.33, 0.5, 1, and 2 were prepared by a modified sol–gel route using tetradecyltrimethylammonium bromide as templating agent and acetic acid as precipitation agent. To obtain the molar ratios mentioned above, (NH_4_)_6_Mo_7_O_24_·4H_2_O and NH_4_VO_3_ precursor solutions were added in corresponding amounts to a solution of tetradecyltrimethylammonium bromide (0.01 M) and kept under stirring for 30 min at 60 °C. The molar ratio metal:templating agent in all the experiments was 0.3. The resulted sols were then precipitated with acetic acid (pH 3.5–4), separated by filtration and washed thoroughly with distilled water and ethanol [28]. The precipitates were then dried at 100 °C overnight and finally calcined successively at 200 °C, 400 °C, and 550 °C for 2 h at each temperature.

### 2.2. Characterization of the Catalysts

The surface areas of the catalysts were measured from the nitrogen adsorption–desorption isotherms collected with a Micromeritics ASAP 2010 analyzer (Micromeritics, Norcross, GA, USA) at −196 °C using the Brunauer–Emmett–Teller (BET) method. In the first stage, before the measurement 0.5 g of each catalyst sample was outgassed at 150 °C for 12 h. The pore diameter was determined by applying the Barret–Joyner–Haleda (BJH) formalism to the adsorption branch of the isotherms. The total pore volume was determined from the amount adsorbed at a relative pressure of 0.977.

X-ray diffraction (XRD) patterns were collected with a Shimadzu XRD 7000 diffractometer (Shimadzu, Osaka, Japan) using a Ni filter and the Cu*Kα* radiation. The intensity was measured by scanning steps in the 2*θ* ranges 5° to 80°. The mean crystallite size of the samples (expressed in nm) was estimated by applying the Scherrer’s Equation to the XRD line-broadening.

(1) $$d_{XRD}=\frac{k\lambda }{\beta cos\theta }$$

where: *k* is a constant equal to 0.9 (shape factor); *λ* the wavelength of the Cu*Kα* X-ray radiation; *β* the true half-peak width, and *θ* the diffraction angle.

The acidity of the catalysts was determined from the NH_3_-TPD profiles using a chemisorption apparatus (Porotec Instrument, Frankfurt, Germany) equipped with a programmable temperature furnace and thermal conductivity detector (TCD). All catalysts (0.05 g) were treated for 2 h with 40 mL·min^−1^ He at 500 °C. The temperature was increased from ambient to 500 °C with a heating rate of 5 °C·min^−1^ and then lowered to 40 °C. After that, 5% NH_3_/He (10 mL·min^−1^) has been passed for 30 min at 40 °C and purged with 40 mL·min^−1^ He for 1 h. Under the same flow of He, the temperature was raised from 40 °C to 700 °C with 10 °C·min^−1^, and the desorbed ammonia was analyzed.

The UV–VIS spectra were recorded using a UV3600 UV–VIS spectrophotometer (Shimadzu, Osaka, Japan) equipped with a Shimadzu ISR-3100 integrating sphere (Shimadzu, Osaka, Japan) with an incident light angle of 0° to 8°, wavelength range of 220 to 2600 nm and two light sources: D_2_ (deuterium) lamp for the ultraviolet range and WI (halogen) lamp for the visible and near-infrared range, and three detectors, consisting of a PMT (photomultiplier tube) for the ultraviolet and visible regions and InGaAs and cooled PbS detector for the near-infrared region. The spectra were recorded in the range of 220 to 2600 nm (switching the wavelength of the lamps in between 282 nm and 393 nm) with a wavelength step of 2 nm, having the slit width of 8 nm. The UV–VIS spectra were measured using samples diluted with extra pure barium sulfate (purchased from NacalaiTesque, Kyoto, Japan).

Temperature-programmed reduction by hydrogen (H_2_-TPR) was conducted on a chemisorption analyzer (Porotec Instrument, Frankfurt, Germany) equipped with a TCD (Porotec Instrument, Frankfurt, Germany). The mixed molybdena–vanadia samples (50–60 mg) were first outgassed at 200 °C for 1 h in He flow (20 mL·min^−1^) and after that, cooled to room temperature. Then the samples were heated to 700 °C under a 10% H_2_/Ar gas flow (20 mL·min^−1^) at a rate of 5 °C·min^−1^.

X-ray photoelectron spectroscopy measurements (XPS) were carried out in a SPECS surface-science cluster, including an XPS spectrometer equipped with a monochromatic Al K_α1_ X-ray source (photon energy 1486.74 eV) and operating under ultrahigh vacuum at 1.3 × 10^−13^ atm. The photoelectrons were collected by a 150 mm radius Phoibos electron analyzer (Kratos, Manchester, UK) with a pass energy of 30 eV. The binding energies were corrected for the surface-charging effects during the measurements by using the C1s core level (284.6 eV) of the adventitious carbon as an internal reference.

Raman spectra were recorded using an AvaRaman-532-TEC spectrometer (Avantes, Louis Ville, CO, USA) equipped with a 532 nm laser with a rated output of 50 mW, a 1200 mm^−1^ line and an AvaRaman-PRB-532 probe (Avantes, Louis Ville, CO, USA).

### 2.3. Catalytic Tests

Toluene disproportionation was performed in a fixed-bed reactor (i.d., 10 mm) at atmospheric pressure using nitrogen as a carrier gas. Different reaction parameters were assessed during this study, and the general reaction conditions for the evaluation of the catalytic activity were: 0.1 g of catalyst, reaction temperature between 350 and 450 °C, nitrogen flow rate 30 to 70 mL·min^−1^, 1000 to 1500 ppm of toluene.

The selective oxidation reaction of toluene was carried out at atmospheric pressure in a fixed-bed reactor (i.d., 10 mm) using air as an oxidation agent and as a carrier gas as well. The flow rate of air was 50 mL·min^−1^ and the toluene concentration of 1500 ppm.

For both reactions, the analysis of the product was done with a gas chromatograph (Thermo-Quest, Waltham, MA, USA) equipped with a hydrogen flame ionization detector (FID). CO_2_ was analyzed with a Thermo Finnigan Gas-Chromatograph (Thermo Finnigan, Waltham, MA, USA) equipped with a TCD (Thermo Finnigan, Waltham, MA, USA).

The toluene conversion was determined with Formula 2.

(2) $$X_{\mathrm{toluene}}\;=\;\frac{C_{in}-C_{out}}{C_{in}}\times 100$$

where *C_in_* and *C_out_* are the concentration of toluene in the inlet and outlet gas.

The yield of xylenes was calculated with Formula 3.

Y_xylenes_ = (moles of toluene yielded to xylenes/moles of toluene in the inlet gas) × 100 (3)

The selectivity of p-xylene was determined with Formula 4.

S_p-xylene_ = (moles of p-xylene/total moles of xylenes) × 100 (4)

The benzaldehyde selectivity was calculated with Formula 5.

S_benzald_(%) = (moles of toluene yielded to benzaldehyde/total moles of toluene transformed) × 100 (5)

The carbon balance, for all catalytic reactions, has been within reasonable limits with an error of ±5%.

## 3. Results and Discussions

### 3.1. Catalysts Characterization

**Figure 1:**
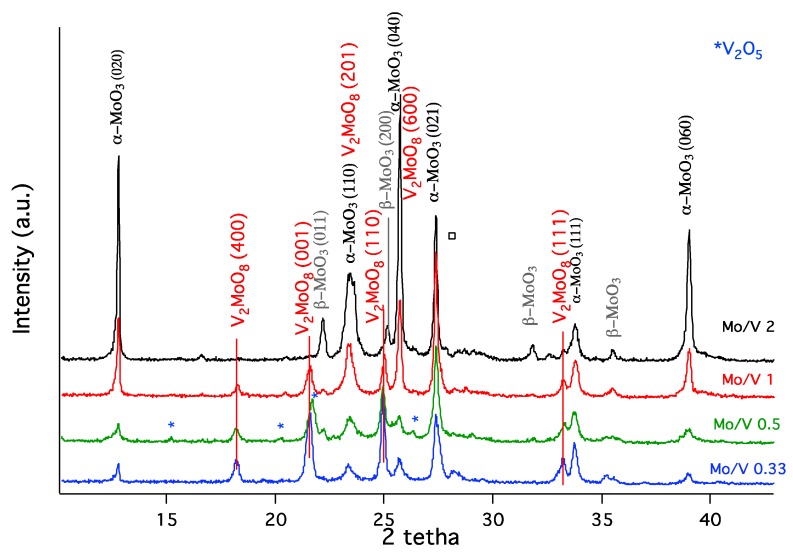
X-ray diffraction (XRD) patterns of mixed molybdena–vanadia catalysts.

The XRD patterns of the mixed molybdena–vanadia catalysts, gathered in Figure 1, show specific diffraction lines of (i) orthorhombic molybdite phase of α-MoO_3_ (JCPDS card no. 76-1003), which consist of layered MoO_6_ octahedra, corner or edge-connected between them [29], (ii) monoclinic phase of β-MoO_3_ (JCPDS card no. 47-1081) containing also corner of edged shared MoO_6_ octahedra arranged this time in a perovskite-like structure [30], and (iii) base-centered monoclinic V_2_MoO_8_ phase (JCPDS card no. 20-1377), which was ascribed to a shear structure of octahedra integrated in a three-dimensional network of corner-linked octahedra [31]. The lines attributed to the orthorhombic shcherbinaite phase of V_2_O_5_ (JCPDS card no. 41-1426) were better observed for the catalyst with the ratio Mo/V = 0.5. For the catalysts with Mo/V = 0.33 and Mo/V = 0.5, V_2_MoO_8_ represents the dominant phase, while the increase of the Mo loading (Mo/V = 2 and Mo/V = 1) led to an increase of the content in both the α-MoO_3_ and β-MoO_3_ phases that occurs in the detriment of V_2_MoO_8_. In addition, the increase of the content in Mo loading led to a higher degree of crystallinity with bigger crystallite sizes (from 31 nm, Mo/V = 0.33 to 52 nm, Mo/V = 2), which was also observed for other vanadia like materials containing Mo [32].

Another interesting feature revealed by the Mo/V = 2 sample is the co-existence of the monoclinic β-MoO_3_ with α-MoO_3_ phase mainly orientated in the (0X0) direction ((020), (040), and (060) planes). This is quite unusual behavior for α-MoO_3_ crystals, which are obviously highly anisotropic. Such a phase growth has also been reported for MoO_3_ prepared via physical methods as sputtering [33], pulsed electron beam deposition [34] or the evaporation-induced self-assembly process [35]. For Mo/V ratio of 1, the anisotropic α-MoO_3_ character is re-established. To sum it up, for the formation of a mixt phase, containing V and Mo, a certain Mo/V ratio is needed (i.e., <0.5).

From the adsorption–desorption isotherms, the surface areas of the samples, calcined at 550 °C, were determined. For all studied samples these are very small, i.e., between 0.6 and 2.2 m^2^·g^−1^, due probably to the relatively high calcination temperature and, therefore, we cannot evidence a direct effect of the Mo/V ratio on this parameter.

**Figure 2:**
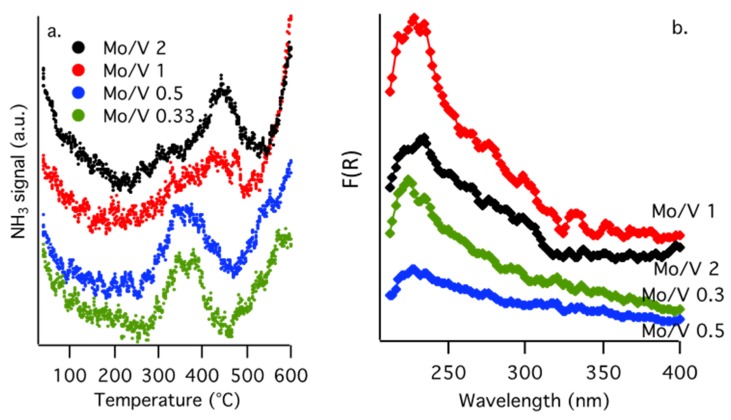
(**a**) NH_3_-TPD profiles for mixed molybdena–vanadia catalysts, (**b**) Diffuse reflectance UV–VIS spectra of molybdenum–vanadium mixed oxides.

The density and strength of the acid sites of the Mo/V samples have been determined by the use of NH_3_-TPD. The profiles were compared in Figure 2a, while the quantities of NH_3_ desorbed are presented in Table 1. As expected, the molar ratio Mo/V influence the acidic properties and NH_3_-TPD shows a bimodal desorption profile with maxima at 350 and 450 °C corresponding to medium and strong acid sites, respectively.

The broad desorption pattern indicates a large distribution of different types of acid sites. The Mo/V molar ratio affects the number, strength and type of the acid sites (Table 1). Lower Mo/V molar ratios induce the formation of moderate acid sites, whereas an increased molybdenum loading led to stronger acid sites as revealed by their desorption temperatures (with maxima at 340 °C for Mo/V = 0.33 and 442 °C for Mo/V = 2, respectively). This behavior is in concordance with previous reports [36] indicating an increase of the strength of the acidity with the molybdenum loading. However, the total amount of the acid sites (μmol·g^−1^) increased with the decreasing of the Mo content in the following order: Mo/V = 2 (3.8) < Mo/V = 1 (5.6) < Mo/V = 0.5 (6.9) < Mo/V = 0.33 (7.8).

Furthermore, these results can be used to distinguish between Brønsted or Lewis sites. As reported in literature, the low-temperature desorption peak, below 350 °C, corresponds to the NH_4_^+^ desorbed from Brønsted acid sites, while the Lewis acid–coordinated sites correspond to NH_3_ desorbed at higher temperature [37]. In this regard, it is obvious that the catalysts with low Mo/V ratios present higher amounts of Brønsted acid sites, while by increasing the Mo loading the amount of Lewis type sites became preponderant.

Correlating these results found in NH_3_-TPD measurements with the phases identified in XRD, we can assume that the addition of Mo, together with the formation of MoO_3_ phases, is strongly related to the presence of the strong acid sites, while the presence of V in higher amounts favors the formation of V_2_MoO_8_ phase that is providing moderate acidity in the material. Obviously, a mixture of the two phases will supply a mixture of moderate and strong acid sites in different proportion on the material.

The UV–VIS spectra of molybdena–vanadia catalysts are shown in Figure 2b. The bands at 210 to 250 nm are attributed to the tetrahedral molybdate species [38], the bands in the region of 290 to 320 nm to octahedrally coordinated Mo^6+^ [39], and the band at 320 nm to the Mo–O–Mo bridge bond of the octahedral coordination [40]. The bands at around 240 and 280 nm correspond to the charge transfer from O^2−^ to V^5+^ tetrahedrally coordinated [41] while that at 350 nm suggests the presence of polymeric vanadium species (V–O–V). For samples containing higher molybdenum loading bands appear in the region of 300 to 330 nm which indicate an interaction between molybdenum and vanadium [42]. These findings are in good agreement with the phases observed in XRD.

**Figure 3:**
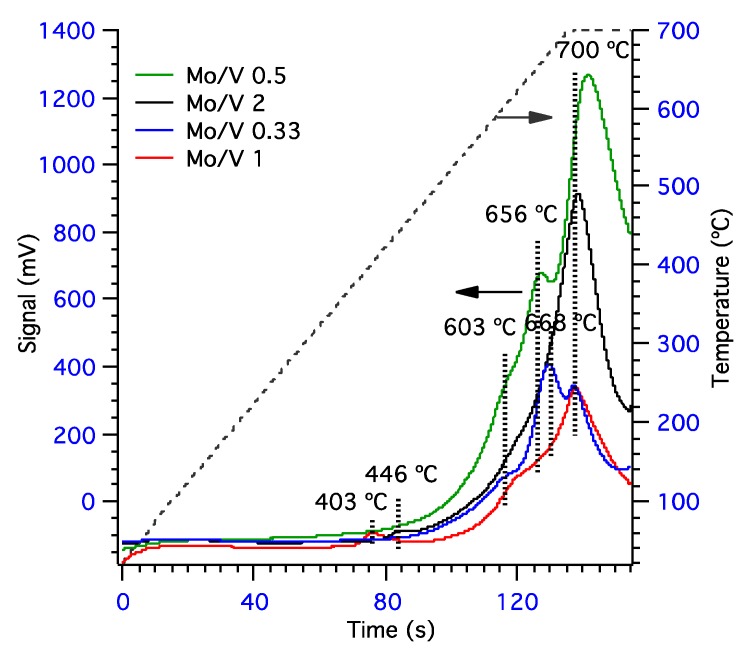
Temperature-programmed reduction by hydrogen (TPR-H_2_) profiles for mixed molybdena–vanadia catalysts.

With the scope to have more information about the reducibility of our samples, temperature programmed reduction in hydrogen flow was conducted (Figure 3). The samples Mo/V = 2 and Mo/V = 1, with the highest amount of Mo, present one reduction peak at low temperature. This reduction peak can be associated with the reduction of octahedrally coordinated Mo^6+^ to Mo^4+^ [43], but it is also possibly due to the reduction of isolated V_2_O_5_ to V_2_O_3_ [44,45,46]. Even though V_2_O_5_ was not evidenced in XRD, isolated V^5+^ species cannot be excluded for the samples Mo/V 2 and 1. However, all samples present a shoulder at ~600 °C, which can also be related to the reduction of V^5+^ from the surface. In the particular case, for Mo/V = 1 sample, the reduction peak corresponding to V^5+^ reduction is shifted to higher values (627 °C) and it is broader, indicating a higher interaction of the V^5+^ with its neighbors and/or possibly the existence of two overlapping peaks (V_2_O_5_ and V_2_MoO_8_). Higher temperature reduction peaks, in the range of 650 to 670 °C, shown only for the sample with high V content (Mo/V = 0.33 and 0.5) can be correlated with the reduction of V^5+^ species strongly interacting with Mo in the V_2_MoO_8_ phase reduction [43]. It is worth mentioning that this phase was evidenced mainly in the sample with a high V content (from XRD), although this phase is also present in the Mo/V = 1 sample. The reduction peak at 700 °C, present in all samples, could be correlated with the octahedral Mo^4+^ to Mo^0^ reduction as well the contribution of the reduction of the tetrahedral molybdenum species Mo^6+^ to Mo^4+^ [47], either from V_2_MoO_8_ or MoO_3_ phases. Both configurations were evidenced by the UV–VIS analysis.

It can be observed that samples containing Mo/V = 0.5 and 2 possess the highest total hydrogen up-take, which indicates an increased amount of reducible lattice oxygen for these two samples. The highest amount of H_2_ uptake (1.56 mmol·m^−2^, Table 2) was found for Mo/V = 0.5 sample and is in correlation with its observed phases in XRD, which also revealed the presence of V_2_O_5_ crystallites only for this sample. Moreover, for the sample with the highest V loading (Mo/V = 0.33) present the lowest hydrogen up-take, meaning that in this case the V–O bonding is more stable and harder to reduce [48].

The H_2_ uptake of the low-temperature peak corresponding to the reduction of octahedrally coordinated Mo^6+^ is lower than the H_2_ uptake from the high-temperature peak, which represents the contribution of both octahedral Mo^4+^ to Mo^0^ and tetrahedral Mo^6+^ to Mo^4+^ reduction. Therefore, the high-temperature peak can be mainly attributed to tetrahedral molybdate species which is in good agreement with DR–UV–VIS data. Additionally, it can be inferred that the high content molybdenum catalysts favor the generation of the tetrahedral molybdate species, which are more reducible than the octahedral ones.

**Figure 4:**
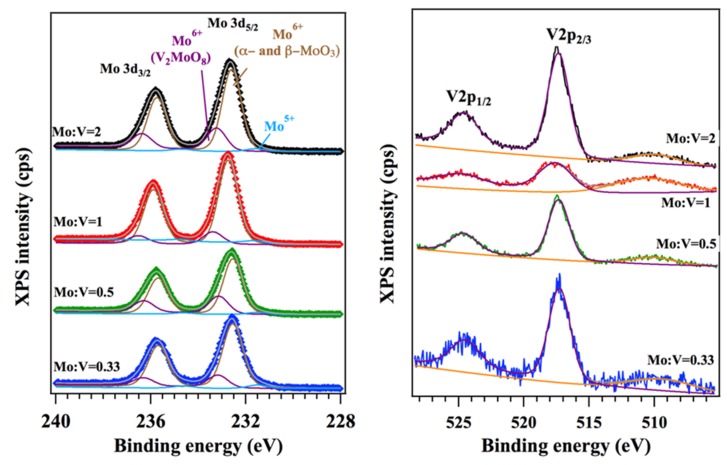
The X-ray photoelectron spectroscopy (XPS) Mo 3d and V 2p regions for mixed molybdena–vanadia catalysts.

XPS results are presented in Figure 4 and Table 3. The binding energies (BE) of Mo 3d, V 2p, and O 1s peaks are representative for Mo^5+^, Mo^6+^, V^5+^, and of O^2−^ in transition metal oxides. The Mo 3d spectrum is composed of three components: one main peak resolved at ~232.5 eV (Mo 3d5/2) corresponding to Mo^6+^ of MoO_3_ [49,50,51] denoted as C1, a second one to Mo^6+^ as a component of V_2_MoO_8_ at ~233.2 eV denoted as C2, and a third doublet shifted to lower binding energy (~231.5 eV) corresponding to a reduced species of Mo^5+^, (~3%, as component of to all Mo-based catalysts) [51] denoted as C3. V 2p binding energy (~517.3 eV) found in all investigated samples correspond to V^5+^ oxidation state, in either V_2_MoO_8_ or V_2_O_5_ species. The oxidation states of the cations identified by XPS are in good agreement with XRD data.

The comparison of XPS with the analytic Mo/V ratios has shown a superficial increase in the Mo species, at the surface, for all the samples. This may also be associated with the agglomeration tendency of vanadium species [52] in the subsurface.

**Figure 5:**
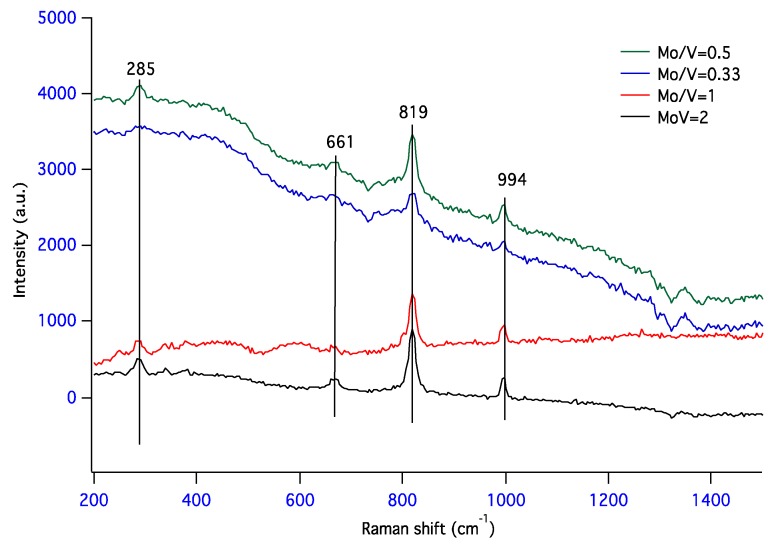
Raman spectra for mixed molybdena–vanadia catalysts.

Additional information regarding the structure of the molybdenum–vanadium mixed oxides was obtained using Raman spectroscopy. Figure 5 presents the Raman spectra of the studied samples with different Mo/V ratios. Irrespective to the Mo/V ratio, all spectra are presenting three Raman peaks: (i) a sharp peak at 994 cm^−1^ that can be assigned to stretching vibration of Mo=O mono-oxo molybdenum oxide species [53] or to hydrated decavanadate species [54]; (ii) a very well-defined peak at 819 cm^−1^ originated from stretching vibrations of Mo–O–Mo groups, and (iii) a weak peak at 661 cm^−1^ characteristic to the vibrations of Mo_2_O_2_ units formed by edge shared MoO_6_ octahedra from α-MoO_3_. In the samples with high vanadium loading (Mo/V = 0.33 and 0.5) the presence of a small band at around 775 cm^−1^ can be observed, which is related to the presence of V–O–Mo vibrations for molybdovanadate species [55] from V_2_MoO_8_. The existence of this phase was also evidenced by XRD analysis. The bands in the low Raman shift region are indicative of the presence of hydrated decavanadate species [54] or the presence of trace amounts of V_2_O_5_ [56]. No band at ~1030 cm^−1^ was evidenced in Raman spectra, which indicates that no isolated vanadia species or V=O stretching modes of surface vanadia are present in these samples, irrespective to Mo/V ratio.

### 3.2. Toluene Disproportionation

**Figure 6:**
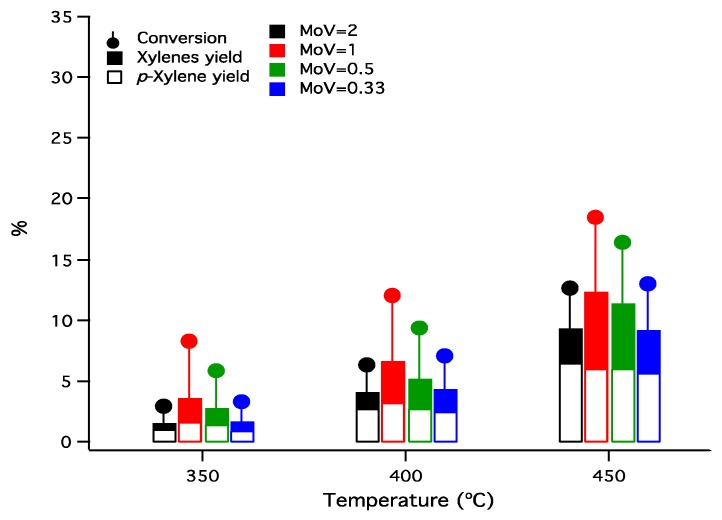
Variation of the conversion of toluene, yields to xylenes and yields to *p*-xylene as function of temperature (N_2_/toluene molar ratio 4, N_2_ flow rate 50 mL·min^−1^, catalyst weight 0.1 g).

Toluene conversion by disproportionation and yields of xylenes as a function of temperature over mixed molybdena–vanadia catalysts are presented in Figure 6. The main reaction products were xylene isomers (p-xylene and o-xylene), benzene, and to a small extent, trimethylbenzene.

As expected, the conversion of toluene increased with the reaction temperature from 350 to 450 °C and is varying with the Mo/V ratio. The highest conversion was obtained on the catalyst Mo/V = 1, while higher amounts of molybdenum led to a drop in the conversion. Summarizing, the toluene conversion varies in the following order Mo/V = 1 > Mo/V = 0.5 > Mo/V = 0.33 > Mo/V = 2 for all reaction temperatures investigated.

The yield to xylenes gathered in Figure 6 increases with the reaction temperature for all samples. The highest yield in xylenes (13%) was obtained for Mo/V = 1 catalyst at 450 °C. Regardless of the reaction temperature, the xylenes yield increased in the following order: Mo/V =2 < Mo/V = 0.33 < Mo/V = 0.5 < Mo/V = 1, the same order as the toluene conversion. The yield in p-xylene is depicted in Figure 6, showing that its production follows, in general, the total yield to xylenes profile.

**Figure 7:**
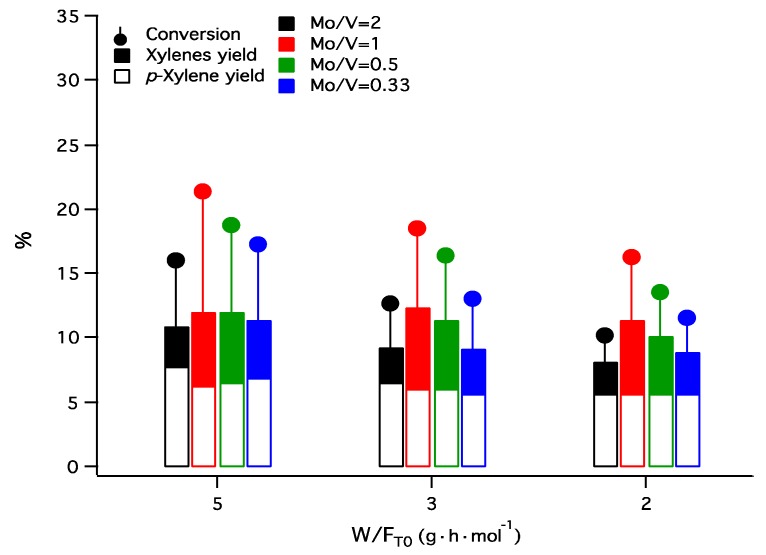
Variation of conversion of toluene and the yield to xylenes and to *p*-xylene as a function of W/F_Tο_ (temperature 450 °C).

The effect of W/F_Tο_ on toluene conversion and yield to xylenes is presented in Figure 7 (W—weight of catalysts, F_Tο_—inlet molar flow rate of toluene). For the same temperature (450 °C), the conversion of toluene increased with W/F_T0_ for all Mo/V ratio. The conversion and yield to xylenes reached maximum over Mo/V = 1, irrespective to the W/F_Tο_ ratio. However, the best W/F_Tο_ ratio is 5 g·h·mol^−1^.

**Figure 8:**
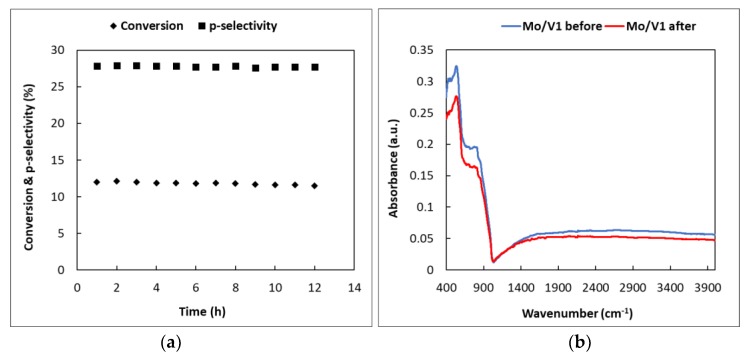
(**a**) The effect of the reaction time on the conversion of toluene and selectivity of *p*-xylene over Mo/V = 1 catalyst (temperature 400 °C, N_2_/toluene molar ratio 4, N_2_ flow rate 50 mL·min^−1^, catalyst weight 0.1 g); (**b**) FTIR spectra of the molybdena–vanadia catalyst before and after the catalytic test.

We expected, as presented in the introduction, that the addition of Mo to the catalyst would increase the stability of the material. To prove the stability of our best catalyst, Figure 8a depicts the variation of the conversion of toluene and selectivity to *p*-xylene as a function of the reaction time over the Mo/V = 1 catalyst. The conversion of toluene decreased slowly in time, while the selectivity has not been influenced by the time on stream, indicating a highly stable material. The fact that the catalyst is stable over time was also evidenced by the conservation of the FTIR spectra after the reaction; no coke deposition has been observed (Figure 8b).

### 3.3. Toluene Selective Oxidation

**Figure 9:**
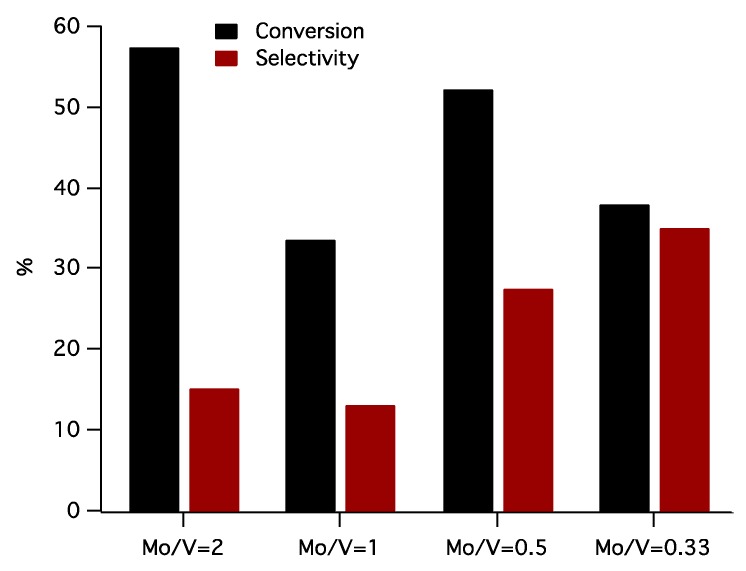
The conversion of toluene and selectivity to benzaldehyde for the oxidation of toluene over molybdena–vanadia catalysts (temperature 450 °C, air flow rate 50 mL·min^−1^, toluene concentration 1500 ppm).

The behavior of molybdena–vanadia catalysts in selective oxidation of toluene at 450 °C is shown in Figure 9. One of the very important challenges of selective oxidation reactions is to prevent the over-oxidation of the reaction products stopping the reaction at an intermediate stage. In our study, the main oxidation products were benzaldehyde, benzyl alcohol, and benzoic acid.

The highest toluene conversion by selective oxidation was achieved using Mo/V = 2 as catalyst (58%), and the series varies in the following order Mo/V = 2 > Mo/V = 0.5 > Mo/V = 0.33 > Mo/V = 1. This behavior is opposite compared to that evidenced for the toluene disproportionation, and this is expected behavior, if we consider that the two reactions require different types of active sites (acid-base sites for disproportionation and redox sites for oxidation). Focusing on active sites needed by hydrocarbon oxidation, according to TPR measurements, the highest amount of reducible lattice oxygen is present in the sample Mo/V = 2 and 0.5, meaning a high activity of these samples, which are in perfect correlation with our results presented in Figure 9, (58% and 51%, respectively). Moreover, it is also well known that a catalyst with an active lattice oxygen can be detrimental to the selectivity in hydrocarbons oxidation due to an advanced oxidation of substrates [57,58,59,60], and this can explain why the best catalyst (in terms of conversion), Mo/V = 2 failed to provide a good selectivity also.

The best selectivity in benzaldehyde (34%) was achieved for Mo/V = 0.33, whereas a decrease of vanadium content induces a downfall in the selectivity of benzaldehyde. In concordance to the characterization results (XRD, XPS), good selectivities in benzaldehyde for Mo/V = 0.5 and Mo/V = 0.33 catalysts could be associated to the presence of the V–O–Mo species from the V_2_MoO_8_ phase, that were evidenced by Raman spectroscopy. In addition, the hydrogen up-takes for these sample sustains the above statements, and revealed that the best selective catalysts are only the samples containing V^5+^ interacting to Mo^6+^ species belonging to V_2_MoO_8_ phases, which, according to other studies [61], seems to be responsible for the high selectivity in benzaldehyde.

### 3.4. General Assessments

**Scheme 1:**
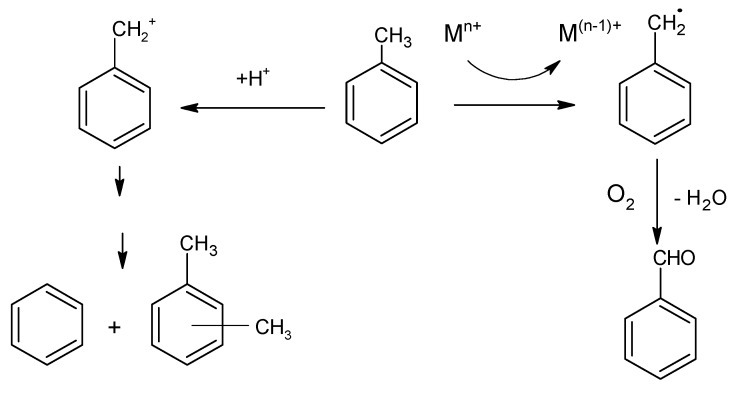
Toluene disproportionation and selective oxidation pathways.

The disproportionation and selective oxidation of toluene investigated in this study indicate that these reactions require different types of active sites on the molybdena–vanadia catalysts (see Scheme 1).

Our results are in line with the results of Hutchings et al. [62] that also found that the oxidation and disproportionation reactions occur onto different active sites on the supported gold–palladium catalysts: metal sites are active for the oxidation reaction and the metal-support interface sites for the disproportionation reaction.

**Figure 10:**
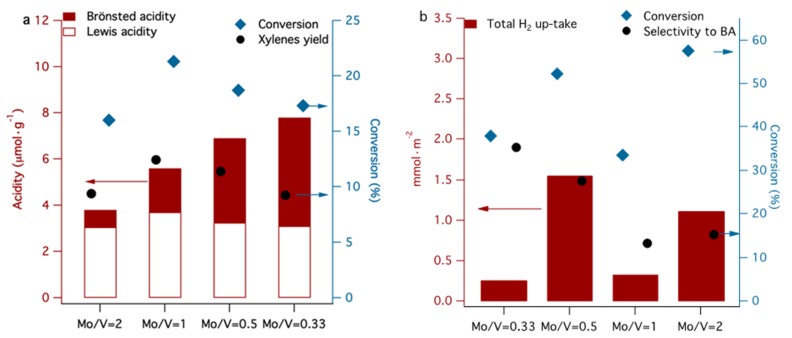
Variation of the conversion of toluene by disproportionation and p-xylene yield (**a**) and toluene conversion by selective oxidation to benzaldehyde selectivity (**b**) as a function of Mo/V ratio.

Figure 10 summarizes the variation of the conversion of toluene by disproportionation and selective oxidation as a function of accessible acid and redox sites. One can observe from Figure 10a, that the disproportionation of toluene is dependent on the amount of Lewis acidity, although the total amount of acidity assists the reaction in terms of both conversion and selectivity. The presence of both phases V_2_MoO_8_ and MoO_3_ are necessary to generate a suitable amount and type of acidity to induce the toluene disproportionation.

The Mo/V = 2 catalyst is the most active in a toluene oxidation reaction and less active in disproportionation reaction, indicating that the same molecule can be activated differently, function of active sites presented on the surface, structural properties and on the reaction conditions. The α-MoO_3_ and β-MoO_3_ phases dominate in the Mo/V = 2 catalyst and provide the highest uptake of H_2_ (see TPR results), indicating higher mobility of lattice oxygen. This suggests that the reducibility represent an important parameter for the selective oxidation reaction.

Despite its high activity in toluene oxidation, Mo/V = 2 does not present the best selectivity, due to its high number of reducible lattice oxygen, which leads to deep oxidation and, therefore, the selectivity toward a specific compound cannot be achieved.

The activation of two molecules of toluene for disproportionation occurs better on Mo/V = 1 catalyst which contains 50–50% molybdenum and vanadium and the XRD data indicate the presence of a mixture of β-MoO_3_ and α-MoO_3_ phases in co-existence with V_2_MoO_8_, which can be correlated with the presence of both medium and strong acid sites. Thus, the strong synergistic behavior of the 50–50% mixture of vanadium and molybdenum oxide phases induce a moderate acidity, which meets all conditions for high catalytic activity in toluene disproportionation [63]. Conversely, a high content of V_2_MoO_8_ phase is more convenient if a high selectivity in toluene oxidation is desired, as is observed for the catalyst with Mo/V = 0.33. By simply estimating the catalyst activity and the product distribution, one can have an idea of the relative implication of acid and redox types of sites. Moreover, the disproportion of toluene was proposed recently as a model reaction for obtaining valid information on the characteristics of the active acid sites of solid catalysts [64].

## 4. Conclusions

Molybdenum–vanadium mixed oxides with different Mo/V ratios are prepared by a modified method. Depending on the chemical composition, the X-ray diffraction indicates the formation of mixtures of different phases. The relative composition of these phases is related to the acidic/oxidative properties of these materials.

Thus, toluene disproportionation requires large quantities of strong acid sites. A higher yield in xylenes was obtained using Mo/V = 1, a catalyst for which the presence of the mixture of α-MoO_3_ phases orientated in the direction (020), (040), and (060) generates specific catalytic sites affording this reaction with good selectivity. Conversely, the catalyst Mo/V = 0.33 in which the quantity of vanadium is increased to 23%, favors the formation of V_2_MoO_8_ as a dominant phase, helpful to skip deep oxidation of toluene and to allow a good selectivity to benzaldehyde.

The proper ratio between Mo and V is a prerequisite to achieving the right proportion between the active and selective sites, thus, enabling a high selectivity to benzaldehyde or to *p*-xylenes.

## Figures and Tables

**Table 1:** Temperature programmed desorption of NH_3_ for mixed molybdena–vanadia catalysts.

Mo/V Molar Ratio	T_des_ Range (°C)	NH_3_ Desorbed (μmol·g^−1^)	Weak Acidity (μmol·g^−1^)	Strong Acidity (μmol·g^−1^)
0.33	280–450	7.8	4.7	3.1
0.5	280–450	6.9	3.65	3.25
1	310–500	5.6	1.9	3.7
2	290–520	3.8	0.75	3.05

**Table 2:** H_2_-Temperature programmed reduction for mixed molybdena–vanadia catalysts.

Mo/V Molar Ratio	T_des_ (°C)				H_2_ Uptake (mmol·m^−2^)				Total H_2_ Uptake (mmol·m^−2^)
	LT	HT1	HT*2*	HT*3*	LT	HT1	HT*2*	HT*3*	
0.33	-	603	668	700	-	0.01	0.16	0.10	0.26
0.5	-	603	656	700	-	0.12	0.32	1.13	1.56
1	403	627	-	700	0.05	0.03	-	0.25	0.33
2	446	603	-	700	0.02	0.03	-	0.83	0.88

**Table 3:** X-ray photoelectron spectroscopy (XPS) binding energies for Mo 3d and V 2p for mixed molybdena–vanadia catalysts.

Mo/V Molar Ratio	Mo 3d_5/2_ (BE, eV)			V 2p_3/2_ (BE, eV)	Mo/V Atomic Ratio (XPS)
	C_1_ Mo^5+^	C_2_ Mo^6+^(MoO_3_)	C_3_ Mo^6+^(V_2_MoO_8_)		
0.33	231.5	232.5	233.2	517.3	2
0.5	231.5	232.5	233.2	517.3	3
1	231.5	232.7	233.4	517.6	5
2	231.5	232.6	233.2	517.3	7

## References

[1] Ji Y.-J., Zhang B., Xu L., Wu H., Peng H., Chen L., Liu Y., Wu P. (2011). Core/shell-structured Al-MWW@B-MWW zeolites for shape-selective toluene disproportionation to para-xylene. J. Catal..

[2] Tsai T.-C., Chen W.-H., Lai C.S., Liu S.-B., Wang I., Ku C.S. (2004). Kinetics of toluene disproportionation over fresh and coked H-mordenite. Catal. Today.

[3] Uguina M.A., Sotelo J.L., Serrano D.P. (1993). Roles of ZSM-5 modifier agents in selective toluene disproportionation. Can. J. Chem. Eng..

[4] Kunieda T., Kim J.-H., Niwa M. (1999). Source of Selectivity of p-Xylene Formation in the Toluene Disproportionation over HZSM-5 Zeolites. J. Catal..

[5] Xiong Y., Rodewald P.G., Chang C.D. (1995). On the Mechanism of Toluene Disproportionation in a Zeolite Environment. J. Am. Chem. Soc..

[6] Fong Y.Y., Abdullah A.Z., Ahmad A.L., Bhatia S. (2008). Development of functionalized zeolite membrane and its potential role as reactor combined separator for para-xylene production from xylene isomers. Chem. Eng. J..

[7] Gallego E.M., Portilla M.T., Paris C., León-Escamilla A., Boronat M., Moliner M., Corma A. (2017). “Abinitio” synthesis of zeolites for preestablished catalytic reactions. Science.

[8] Cejka J., Wichterlová B., Eder-Mirth G., Lercher J.A. (1996). Decisive role of transport rate of products for zeolite para-selectivity: Effect of coke deposition and external surface silylation on activity and selectivity of HZSM-5 in alkylation of toluene. Zeolites.

[9] Suganuma S., Nakamura K., Okuda A., Katada N. (2017). Enhancement of catalytic activity for toluene disproportionation by loading Lewis acidic nickel species on ZSM-5 zeolite. Mol. Catal..

[10] Mitra B., Kunzru D. (2013). Enhancing p-xylene productivity for disproportionation of toluene in microstructured reactors. Chem. Eng. Process. Process Intensif..

[11] Tsai T.-C. (2006). Reactivation of acidic sites in mordenite used in toluene disproportionation. Appl. Catal. A.

[12] Hino M., Kurashige M., Matsuhashi H., Arata K. (2006). A solid acid of tungsta-niobia more active than aluminosilicates for decompositions of cumene, ethylbenzene, and toluene. Appl. Catal. A.

[13] Wang D., Osmundsen C.M., Taarning E., Dumesic J.A. (2013). Selective Production of Aromatics from Alkylfurans over Solid Acid Catalysts. ChemCatChem.

[14] Williams C.L., Chang C.-C., Do P., Nikbin N., Caratzoulas S., Vlachos D.G., Lobo R.F., Fan W., Dauenhauer P.J. (2012). Cycloaddition of Biomass-Derived Furans for Catalytic Production of Renewable p-Xylene. ACS Catal..

[15] Huang M.M., Howe R.F., Bibby D.M., Chang C.D., Howe R.F., Yurchak S. (1988). Reactions of Methanol and Toluene over Molybdenum Zeolites. Studies in Surface Science and Catalysis.

[16] Al-Khattaf S., Musilová-Pavlačková Z., Ali M.A., Čejka J. (2008). Comparison of Activity and Selectivity of SSZ-33 Based Catalyst with other Zeolites in Toluene Disproportionation. Top. Catal..

[17] Kesavan L., Tiruvalam R., Rahim M.H.A., bin Saiman M.I., Enache D.I., Jenkins R.L., Dimitratos N., Lopez-Sanchez J.A., Taylor S.H., Knight D.W. (2011). Solvent-Free Oxidation of Primary Carbon-Hydrogen Bonds in Toluene Using Au-Pd Alloy Nanoparticles. Science.

[18] Losada P.J., Heckl I., Bertok B., Friedler F., García-Ojeda J., Argoti A. (2015). Process Network Synthesis for Benzaldehyde Production: P-graph Approach. Chem. Eng. Trans..

[19] Xue M., Chen H., Zhang H., Auroux A., Shen J. (2010). Preparation and characterization of V-Ag-O catalysts for the selective oxidation of toluene. Appl. Catal. A.

[20] Ge J., Xue M., Sun Q., Auroux A., Shen J. (2007). Surface acidic and redox properties of V-Zr-O catalysts for the selective oxidation of toluene to benzaldehyde. J. Mol. Catal. A Chem..

[21] Bautista F.M., Campelo J.M., Luna D., Luque J., Marinas J.M. (2007). Gas-phase selective oxidation of toluene on TiO_2_–sepiolite supported vanadium oxides: Influence of vanadium loading on conversion and product selectivities. Catal. Today.

[22] Solsona B., Dejoz A., Garcia T., Concepción P., Nieto J.M.L., Vázquez M.I., Navarro M.T. (2006). Molybdenum–vanadium supported on mesoporous alumina catalysts for the oxidative dehydrogenation of ethane. Catal. Today.

[23] Heracleous E., Machli M., Lemonidou A.A., Vasalos I.A. (2005). Oxidative dehydrogenation of ethane and propane over vanadia and molybdena supported catalysts. J. Mol. Catal. A Chem..

[24] Liu R., Wang T., Cai D., Jin Y. (2014). Highly Efficient Production of Acrylic Acid by Sequential Dehydration and Oxidation of Glycerol. Ind. Eng. Chem. Res..

[25] Nedyalkova R., Ilieva L., Bernard M.C., Hugot-Le Goff A., Andreeva D. (2009). Gold supported catalysts on titania and ceria, promoted by vanadia or molybdena for complete benzene oxidation. Mater. Chem. Phys..

[26] Tokarz-Sobieraj R., Witko M., Gryboś R. (2005). Reduction and re-oxidation of molybdena and vanadia: DFT cluster model studies. Catal. Today.

[27] Vishnetskaya M.V., Tomskiy I.S. (2011). Role of Singlet Oxygen in the Oxidation of Toluene on Vanadium Molybdenum Catalytic Systems. Chem. Sustain. Dev..

[28] Li Y., Liu T., Li T., Peng X. (2015). Hydrothermal fabrication of controlled morphologies of MoO_3_ with CTAB: Structure and growth. Mater. Lett..

[29] Huang P.-R., He Y., Cao C., Lu Z.-H. (2014). Impact of lattice distortion and electron doping on α-MoO_3_ electronic structure. Sci. Rep..

[30] Rao M.C., Ravindranadh K., Kasturi A., Shekhawat M.S. (2013). Structural Stoichiometry and Phase Transitions of MoO_3_ Thin Films for Solid State Microbatteries. Res. J. Recent Sci..

[31] Giebeler L., Kampe P., Wirth A., Adams A.H., Kunert J., Fuess H., Vogel H. (2006). Structural changes of vanadium–molybdenum–tungsten mixed oxide catalysts during the selective oxidation of acrolein to acrylic acid. J. Mol. Catal. A Chem..

[32] Adams A.H., Haaß F., Buhrmester T., Kunert J., Ott J., Vogel H., Fuess H. (2004). Structure and reaction studies on vanadium molybdenum mixed oxides. J. Mol. Catal. A Chem..

[33] Zou J.Y., Schrader G.L. (1998). Deposition of multiphase molybdate thin films by reactive sputtering. Thin Solid Films.

[34] Liu C., Li Z., Zhang Z. (2011). Growth of [010] oriented α-MoO_3_ nanorods by pulsed electron beam deposition. Appl. Phys. Lett..

[35] Brezesinski T., Wang J., Tolbert S.H., Dunn B. (2010). Ordered mesoporous α-MoO_3_ with iso-oriented nanocrystalline walls for thin-film pseudocapacitors. Nat. Mater..

[36] Bhaskar T., Reddy K.R., Kumar C.P., Murthy M.R.V.S., Chary K.V.R. (2001). Characterization and reactivity of molybdenum oxide catalysts supported on zirconia. Appl. Catal. A.

[37] Wang D., Jangjou Y., Liu Y., Sharma M.K., Luo J., Li J., Kamasamudram K., Epling W.S. (2015). A comparison of hydrothermal aging effects on NH3-SCR of NOx over Cu-SSZ-13 and Cu-SAPO-34 catalysts. Appl. Catal. B.

[38] Ciambelli P., Sannino D., Palma V., Vaiano V., Eloy P., Dury F., Gaigneaux E.M. (2007). Tuning the selectivity of MoOx supported catalysts for cyclohexane photo oxidehydrogenation. Catal. Today.

[39] Vakros J., Lycourghiotis A., Voyiatzis G.A., Siokou A., Kordulis C. (2010). CoMo/Al_2_O_3_-SiO_2_ catalysts prepared by co-equilibrium deposition filtration: Characterization and catalytic behavior for the hydrodesulphurization of thiophene. Appl. Catal. B.

[40] Duan A., Wan G., Zhao Z., Xu C., Zheng Y., Zhang Y., Dou T., Bao X., Chung K. (2007). Characterization and activity of Mo supported catalysts for diesel deep hydrodesulphurization. Catal. Today.

[41] Higashimoto S., Matsuoka M., Yamashita H., Anpo M., Kitao O., Hidaka H., Che M., Giamello E. (2000). Effect of the Si/Al Ratio on the Local Structure of V Oxide/ZSM-5 Catalysts Prepared by Solid-State Reaction and Their Photocatalytic Reactivity for the Decomposition of NO in the Absence and Presence of Propane. J. Phys. Chem. B.

[42] Mitran G., Pavel O.-D., Marcu I.-C. (2013). Molybdena–vanadia supported on alumina: Effective catalysts for the esterification reaction of acetic acid with n-butanol. J. Mol. Catal. A Chem..

[43] Ghampson I.T., Pecchi G., Fierro J.L.G., Videla A., Escalona N. (2017). Catalytic hydrodeoxygenation of anisole over Re-MoOx/TiO_2_ and Re-VOx/TiO_2_ catalysts. Appl. Catal. B.

[44] Benomar S., Massó A., Solsona B., Issaadi R., López Nieto M.J. (2018). Vanadium Supported on Alumina and/or Zirconia Catalysts for the Selective Transformation of Ethane and Methanol. Catalysts.

[45] Deo G., Wachs I.E. (1994). Reactivity of Supported Vanadium Oxide Catalysts: The Partial Oxidation of Methanol. J. Catal..

[46] Chen K., Bell A.T., Iglesia E. (2002). The Relationship between the Electronic and Redox Properties of Dispersed Metal Oxides and Their Turnover Rates in Oxidative Dehydrogenation Reactions. J. Catal..

[47] Alvarez-Amparán M.A., Cedeño-Caero L. (2017). MoOx-VOx based catalysts for the oxidative desulfurization of refractory compounds: Influence of MoOx-VOx interaction on the catalytic performance. Catal. Today.

[48] Liu X., Duan L., Yang W., Zhu X. (2017). Oxidative dehydrogenation of n-butane to butenes on Mo-doped VMgO catalysts. RSC Adv..

[49] Hussain O.M., Srinivasa Rao K., Madhuri K.V., Ramana C.V., Naidu B.S., Pai S., John J., Pinto R. (2002). Growth and characteristics of reactive pulsed laser deposited molybdenum trioxide thin films. Appl. Phys. A.

[50] Fleisch T.H., Mains G.J. (1982). An XPS study of the UV reduction and photochromism of MoO_3_ and WO_3_. J. Chem. Phys..

[51] Du Y., Li G., Peterson E.W., Zhou J., Zhang X., Mu R., Dohnálek Z., Bowden M., Lyubinetsky I., Chambers S.A. (2016). Iso-oriented monolayer α-MoO_3_(010) films epitaxially grown on SrTiO_3_(001). Nanoscale.

[52] Matralis H.K., Papadopoulou C., Kordulis C., Aguilar Elguezabal A., Cortes Corberan V. (1995). Selective oxidation of toluene over V_2_O_5_/TiO_2_ catalysts. Effect of vanadium loading and of molybdenum addition on the catalytic properties. Appl. Catal. A.

[53] Bañares M.A., Wachs I.E. (2002). Molecular structures of supported metal oxide catalysts under different environments. J. Raman Spectrosc..

[54] Deo G., Wachs I.E. (1991). Predicting molecular structures of surface metal oxide species on oxide supports under ambient conditions. J. Phys. Chem..

[55] Yang S., Iglesia E., Bell A.T. (2005). Oxidative Dehydrogenation of Propane over V_2_O_5_/MoO_3_/Al_2_O_3_ and V_2_O_5_/Cr_2_O_3_/Al_2_O_3_:  Structural Characterization and Catalytic Function. J. Phys. Chem. B.

[56] Bañares M.A., Khatib S.J. (2004). Structure–activity relationships in alumina-supported molybdena–vanadia catalysts for propane oxidative dehydrogenation. Catal. Today.

[57] Rischard J., Antinori C., Maier L., Deutschmann O. (2016). Oxidative dehydrogenation of n-butane to butadiene with Mo-V-MgO catalysts in a two-zone fluidized bed reactor. Appl. Catal. A.

[58] Téllez C., Abon M., Dalmon J.A., Mirodatos C., Santamaría J. (2000). Oxidative Dehydrogenation of Butane over VMgO Catalysts. J. Catal..

[59] Harlin M.E., Niemi V.M., Krause A.O.I. (2000). Alumina-Supported Vanadium Oxide in the Dehydrogenation of Butanes. J. Catal..

[60] Lee H., Lee J.K., Hong U.G., Yoo Y., Cho Y.-J., Lee J., Jang H.-S., Jung J.C., Song I.K. (2012). Effect of oxygen capacity and oxygen mobility of supported Mg_3_(VO_4_)_2_ catalysts on the performance in the oxidative dehydrogenation of n-butane. J. Ind. Eng. Chem..

[61] Guerrero-Pérez M.O., Herrera M.C., Malpartida I., Larrubia M.A., Alemany L.J. (2006). Characterization and FT-IR study of nanostructured alumina-supported V-Mo-W-O catalysts. Catal. Today.

[62] Cao E., Sankar M., Nowicka E., He Q., Morad M., Miedziak P.J., Taylor S.H., Knight D.W., Bethell D., Kiely C.J. (2013). Selective suppression of disproportionation reaction in solvent-less benzyl alcohol oxidation catalysed by supported Au–Pd nanoparticles. Catal. Today.

[63] Kubů M., Žilková N., Zones S.I., Chen C.-Y., Al-Khattaf S., Čejka J. (2016). Three-dimensional 10-ring zeolites: The activities in toluene alkylation and disproportionation. Catal. Today.

[64] Guisnet M., Pinard L. (2018). Characterization of acid-base catalysts through model reactions. Catal. Rev..

